# Response inhibition of cigarette-related cues in male light smokers: behavioral evidence using a two-choice oddball paradigm

**DOI:** 10.3389/fpsyg.2015.01506

**Published:** 2015-10-15

**Authors:** Zhao Xin, Liu X. Ting, Zan X. Yi, Dai Li, Zhou A. Bao

**Affiliations:** ^1^Behavior Rehabilitation Training Research Institution, School of Psychology, Northwest Normal UniversityLanzhou, China; ^2^Lanzhou University Second HospitalLanzhou, China; ^3^School of Psychology, Beijing Normal UniversityBeijing, China

**Keywords:** male light smokers, cigarette-related cues, response inhibition, two-choice oddball paradigm

## Abstract

Behavioral inhibitory control has been shown to play an important role in a variety of addictive behaviors. A number of studies involving the use of Go/NoGo and stop-signal paradigms have shown that smokers have reduced response inhibition for cigarette-related cues. However, it is not known whether male light smokers’ response inhibition for cigarette-related cues is lower than that of non-smokers in the two-choice oddball paradigm. The objective of the current study was to provide further behavioral evidence of male light smokers’ impaired response inhibition for cigarette-related cues, using the two-choice oddball paradigm. Sixty-two male students (31 smokers, 31 non-smokers), who were recruited via an advertisement, took part in this two-choice oddball experiment. Cigarette-related pictures (deviant stimuli) and pictures unrelated to cigarettes (standard stimuli) were used. Response inhibition for cigarette-related cues was measured by comparing accuracy (ACC) and reaction time (RT) for deviant and standard stimuli in the two groups of subjects. An analysis of variance (ANOVA) showed that in all the participants, ACC was significantly lower for deviant stimuli than for standard stimuli. For deviant stimuli, the RTs were significantly longer for male light smokers than for male non-smokers; however, there was no significant difference in RTs for standard stimuli. Compared to male non-smokers, male light smokers seem to have a reduced ability to inhibit responses to cigarette-related cues.

## Introduction

The sense of craving for smoking triggered by cigarette-related cues promotes the maintenance of smoking behavior, thus affecting cigarette withdrawal (Waters et al., 2004). Currently, relevant studies indicate that smokers have impaired response inhibition for cigarette-related cues (Spinella, 2002; Brody et al., 2004; Gallinat et al., 2006; McBride et al., 2006; Xu et al., 2008; Luijten et al., 2011; MacKillop et al., 2011). Response inhibition is an important executive function. It refers to the suppression of inappropriate or no longer relevant behavior, which allows flexible, intentional, behavioral reactions to the environment (Groman et al., 2009; Verbruggen and Logan, 2009). In general, for smokers, cigarette-related cues increase the craving for smoking, which leads to automatic attentional biases to smoking cues (Johnsen et al., 1997; Waters and Feyerabend, 2000; Ehrman et al., 2002). Consequently, the attentional bias toward cigarette-related cues in smokers occupies their cognitive resources and affects their performance on response inhibition tasks (Ryan, 2002; Franken, 2003).

In recent years, studies using event-related potentials (ERPs) have found that cigarette-related cues can impair smokers’ response inhibition (Luijten et al., 2011; Impey et al., 2013; Buzzell et al., 2014; Logemann et al., 2014). Studies have found that compared to controls, reduced NoGo-N2 amplitudes in smokers were accompanied by decreased task performance, whereas no differences between groups were found in the case of P3 amplitudes. This was found to represent a general lack of response inhibition in smokers (Luijten et al., 2011; Buzzell et al., 2014). The studies suggested that NoGo-N2 reflects a top–down inhibition mechanism, that is, it inhibits inappropriate response tendencies before the movement execution (Kok et al., 2004). The inability to inhibit response tendencies during early processing is likely to reflect that smokers are affected by the nicotine intake because of their tendency to inhibit inappropriate responses before movement execution (Luijten et al., 2011). Recent structural and functional brain imaging studies have found that the anterior cingulated cortex exhibited differential activation during exposure to cigarette-related, as opposed to neutral cues, and that sub-regions of the prefrontal cortex showed cue-elicited activation that was modulated by smoking expectancy. In addition, the volume and density of smokers’ gray matter in the dorsolateral prefrontal cortex and ventrolateral prefrontal cortex are significantly lower than those of non-smokers. The volume of gray matter in the left dorsal anterior cingulated cortex of smokers and the density of the gray matter they have in the right cerebellar hemisphere are also lower than those of non-smokers (Brody et al., 2004; Gallinat et al., 2006). Because the dorsolateral prefrontal cortex plays an important role in working memory and other cognitive domains such as information maintaining and processing, researchers believe that structural defects can lead to cognitive defects in smokers (Azizian et al., 2008; Xu et al., 2008).

Previous studies have mostly used Go/NoGo tasks (Spinella, 2002; Evans et al., 2009; Luijten et al., 2011; Impey et al., 2013; Longo et al., 2013; Buzzell et al., 2014; Rass et al., 2014) and stop-signal tasks (Monterosso et al., 2005; Billieux et al., 2010; de Ruiter et al., 2012; Logemann et al., 2014) to investigate response inhibition for cigarette-related cues in smokers. These studies have shown that commission errors and reaction times (RTs), which reflect response inhibition for cigarette-related cues, were significantly high in smokers compared to non-smokers (Spinella, 2002; Billieux et al., 2010; Nestor et al., 2011; de Ruiter et al., 2012). More precisely, Luijten et al. (2011) used cigarette-related and cigarette-unrelated pictures and a Go/NoGo task to compare the response inhibition of moderate smokers and non-smokers for cigarette-related cues, and found that accuracy in response to NoGo stimuli, which reflects suppression ability, was significantly low in moderate smokers compared to non-smokers. In addition, a stop-signal task was used to assess smokers’ response inhibition ability. Logemann et al. (2014) found no difference between smokers and non-smokers with regard to RTs for Go stimuli, whereas the stop signal RT was dramatically longer in smokers than in non-smokers. However, some studies have found that there was no difference between smokers and non-smokers under neutral conditions in commission error and RT using Go/NoGo task and stop-signal task (Monterosso et al., 2005; Evans et al., 2009; de Ruiter et al., 2012; Buzzell et al., 2014; Rass et al., 2014). For example, Buzzell et al. (2014) compared commission error on NoGo trials and omission error on Go trials between smokers and non-smokers using alphabetic Go/No-Go task, it has been found that there was no significant difference in behavioral results. The results of this research are consistent with Evans et al.’s (2009) study, the stimuli were presented for 800 ms in the Go/No-Go task. The entire experiment consisted of 900 trials, 83 of which were NoGo trials. No differences were found between smokers and non-smokers, with regard to performance accuracy in the NoGo trials and RT in the Go trials.

In a stop-signal task, the reaction that is about to be stopped has been under processing, which has already reached a certain level and which is way beyond the level of the processing of a NoGo stimuli. There is evidence that the basal ganglia have an important effect on the launch and inhibition of the action response in stop-signal tasks (Eagle and Robbins, 2003). The basal ganglia, a group of subcortical nuclei in the forebrain, include the caudate nucleus, putamen, globus pallidus, subthalamic nucleus, and substantia nigra (Alexander and Crutcher, 1990). In the nucleus of the basal ganglia, the subthalamic nucleus may be a critical structure involved in the processing of response inhibition (Cohen and Frank, 2009; Ray et al., 2012; Wiecki and Frank, 2013). Aron and Poldrack (2006) studied the relationship between the subthalamic nucleus and stop signal RT; they found that the stronger the subthalamic nucleus activation, the shorter was the stop signal RT of the participants. Research comparing the structure of the brain mechanism of smokers and non-smokers has revealed that compared to non-smokers, the size and density of the gray matter in the thalamus, cerebellum, and nigra are less in smokers than in non-smokers. This is because the competition between reaction and non-reaction occurs in the nucleus of the basal ganglia, which, once stimulated and damaged, influence the response inhibition (Chambers et al., 2006).

Because in a stop-signal task, participants need to stop their response when they see the stop signal, to maintain a high rate of successful inhibition, they have to pay more attention to the stop signal and consciously wait for it. Consequently, the measurement of RT for Go stimuli may be inaccurate (Verbruggen and Logan, 2008). However, in a Go/NoGo task, the participants only have to respond to Go, and not NoGo stimuli; as Go trials require motor responses, and NoGo trials do not, the inhibitory control effects observed in studies using the Go/NoGo paradigm are likely to be contaminated by response-related processes (Yuan et al., 2008). That is, Go/NoGo tasks may not provide an effective behavioral indicator of response inhibition. Therefore, the present study involved the use of a two-choice oddball task that required the participants to respond to both standard and deviant stimuli by pressing different keys as quickly and accurately as possible, rather than only responding to Go stimuli in a Go/NoGo task. A two-choice oddball task requires responses to both standard and deviant stimuli so that the results are not contaminated by motor response-related processes. As a result, the difference between the RTs for deviant and standard stimuli is the behavioral index of response inhibition (Yuan et al., 2008).

In the present study, the two-choice oddball paradigm was used to further investigate the influence of cigarette-related cues on male light smokers’ ability to inhibit responses. The two-choice oddball paradigm was based on the traditional oddball paradigm. The participants were asked to press different buttons quickly and accurately in response to high probability standard stimuli and low probability deviant stimuli. The response to standard stimuli became the dominant response because the standard stimuli were presented more often than deviant stimuli. Consequently, when the deviant stimuli appeared, the participants needed to inhibit the dominant standard stimuli response to perform accurately.

## Materials and Methods

### Participants

As it was difficult to recruit female smokers, 62 male (31 light smokers, 31 non-smokers) undergraduate students were recruited as participants in the experiment. The data of one smoker was not considered because he misunderstood the task. The final group consisted of 30 smokers and 31 non-smokers. As paid volunteers, all the participants signed an informed consent form prior to participating in the experiment. The experiment was approved by the Academic Committee of the School of Psychology, Northwest Normal University, China. The participants also completed questionnaires eliciting demographic information as well as information about their medical and smoking history; further, they completed the Fagerstrom test for Nicotine Dependence (FTND), Beck Depression Inventory (BDI), and the Barratt Impulsiveness Scale-11(BIS-11; **Table 1**).

**Table 1:** Demographic information of smokers and non-smokers (mean and SD).

	Smokers (*n* = 30)	Non-smokers (*n* = 31)
Age	20.77 (2.22)	21.19 (2.36)
Height	175.18 (7.14)	172.48 (5.82)
Weight	132.80 (20.73)	125.29 (17.28)
Cigarettes/day	13.8 (1.31)	0
Fagerström score	2.67 (1.95)	0
Beck Depression Inventory	9.23 (5.55)	8.77 (5.94)
Barratt Impulsiveness Scale	59.33 (5.52)	59.48 (8.15)

Prior to participating in the study, the participants were administered a short questionnaire that was designed for use in this study, and if they fulfilled one or more of certain conditions, they were excluded from the study. These conditions were if they smoked regularly for <2 years; if they were, at that time, under any medications that may affect cognition; if they were suffering from any medical or psychiatric condition that could affect brain function; if they were <18 years; if they had a history of head trauma; if they smoked >1 marijuana cigarette per week; if they consumed >10 standard drinks of alcohol per week; or if they regularly abused substances other than alcohol or marijuana.

At the time of the experiment, the participants were non-deprived smokers. The non-smokers had no current or previous history of cigarette smoking, and the number of ambidextrous (i.e., right- and left-handed) participants was not significant in either of the groups χ^2^ (61) = 0.669, *p* = 0.414. All the participants had normal or corrected-to-normal vision.

### Instruments

#### The Fagerstrom Test for Nicotine Dependence (FTND)

The FTND is a six-item revised version of the Fagerström Tolerance Questionnaire (Heatherton et al., 1991), and it includes six of the Fagerström Tolerance Questionnaire’s eight items. With its high reliability and validity, the FTND has been used in many countries to test nicotine dependence. The six-item questionnaire includes questions such as “How many cigarettes do you smoke a day?” “How soon after waking up do you smoke your first cigarette?” and “Do you smoke if you are so ill that you are in bed for most of the day?” FTND scores range from 0 to 10; higher scores indicate greater nicotine dependence. A score of 1–3 indicates low nicotine dependence, a score of 4–5 indicates moderate dependence, and a score of 6 indicates high dependence. The revised Chinese version of the FTND, which has been shown to be valid and reliable, was used (Huang et al., 2006).

#### The Beck Depression Inventory (BDI)

The BDI is one of the most widely used self-rating scales of depressive symptoms. It not only assesses the presence of depressive symptoms and their degree of severity but also evaluates depression symptoms in control groups. The BDI was developed by Baker in 1961 (Erbauch, 1961), and the revised Chinese version has been shown to have good validity and reliability (Shek, 1990).

#### Barratt Impulsiveness Scale-11 (BIS-11)

The BIS-11 is a self-rating instrument that is often used to assess impulsiveness. It has three factors (Attentional Impulsiveness, Motor Impulsiveness, and Non-planning Impulsiveness) measured by 26 items. Each factor consists of two sub-scales: Attentional Impulsiveness includes attention and cognitive impulsivity, Motor Impulsiveness includes sports impulsivity and perseverance, and Non-planning Impulsiveness includes automation and cognitive complexity. Items are scored based on the frequency of the appearance of symptoms. The scores range from 1 to 4 (1 = hardly/never, 2 = occasionally, 3 = often, 4 = almost always/always), and 11 items are reverse-scored. Higher scores indicate higher levels of impulsiveness (Patton et al., 1995). The Chinese version of the BIS-11 has good reliability and validity (Zhou, 2006).

### Materials

The two-choice oddball paradigm was used to assess inhibition ability. There were two kinds of stimuli: standard stimuli (cigarette-unrelated pictures) and deviant stimuli (cigarette-related pictures). Stimuli were selected from the International Affective Pictures System and the Internet, and included 27 cigarette-unrelated and 27 cigarette-related pictures. Photoshop 7.0 software was used to edit the pictures. To prevent the participants from guessing the objective of the experiment, a colored frame surrounded the pictures (blue frames for cigarette-related pictures and yellow frames for cigarette-unrelated pictures). The participants were told to press a button to indicate whether the frame was blue or yellow. The pictures were assessed by 61 male students (30 light smokers, 31 non-smokers), who were not participants of the present study but who were recruited from the same population of students as were the participants [age: *F*(1,120) = 0.505, *p* = 0.479, η^2^ = 0.004; height: *F*(1,120) = 1.495, *p* = 0.224, η^2^ = 0.012; weight: *F*(1,120) = 1.622, *p* = 0.205, η^2^= 0.013]. These 54 pictures were rated in terms of valence (1 = very pleasant, 9 = very unpleasant), arousal (1 = drowsy, 9 = excited), dominance (1 = picture control, 9 = you own pictures), and their degree of relatedness to cigarettes (1 = picture has nothing to do with cigarettes, 9 = picture is related to cigarettes). The participants who participated in the picture assessment task were not allowed to participate in the main experiment.

The results showed that there was no difference between cigarette-related pictures (valence: *M* = 4.56, SD = 2.15; arousal: *M* = 4.67, SD = 1.99; dominance: *M* = 5.34, SD = 2.05) and cigarette-unrelated pictures (valence: *M* = 5.21, SD = 1.42; arousal: *M* = 4.98, SD = 1.09; dominance: *M* = 5.29, SD = 1.52) with regard to valence [*F*(1,59) = 3.075, *p* = 0.085, η^2^ = 0.049], arousal [*F*(1,59) = 0.955, *p* = 0.332, η^2^= 0.016], and dominance [*F*(1,59) = 0.024, *p* = 0.878, η^2^ < 0.001]. The degree of relatedness to cigarettes was significantly higher for cigarette-related pictures (*M* = 7.45, SD = 1.66) than it was for cigarette-unrelated pictures [*M* = 2.76, SD = 1.48, *F*(1,59) = 184.56, *p* < 0.001, η^2^ = 0.755].

### Task and Procedure

A modified oddball task was used, and the experiment had two blocks of 200 trials. In each block, 170 standard and 30 deviant stimuli (85% vs. 15%, respectively) were presented. In addition, all the pictures were identical in term of size and resolution (210 × 210 pixels).

**FIGURE 1:**
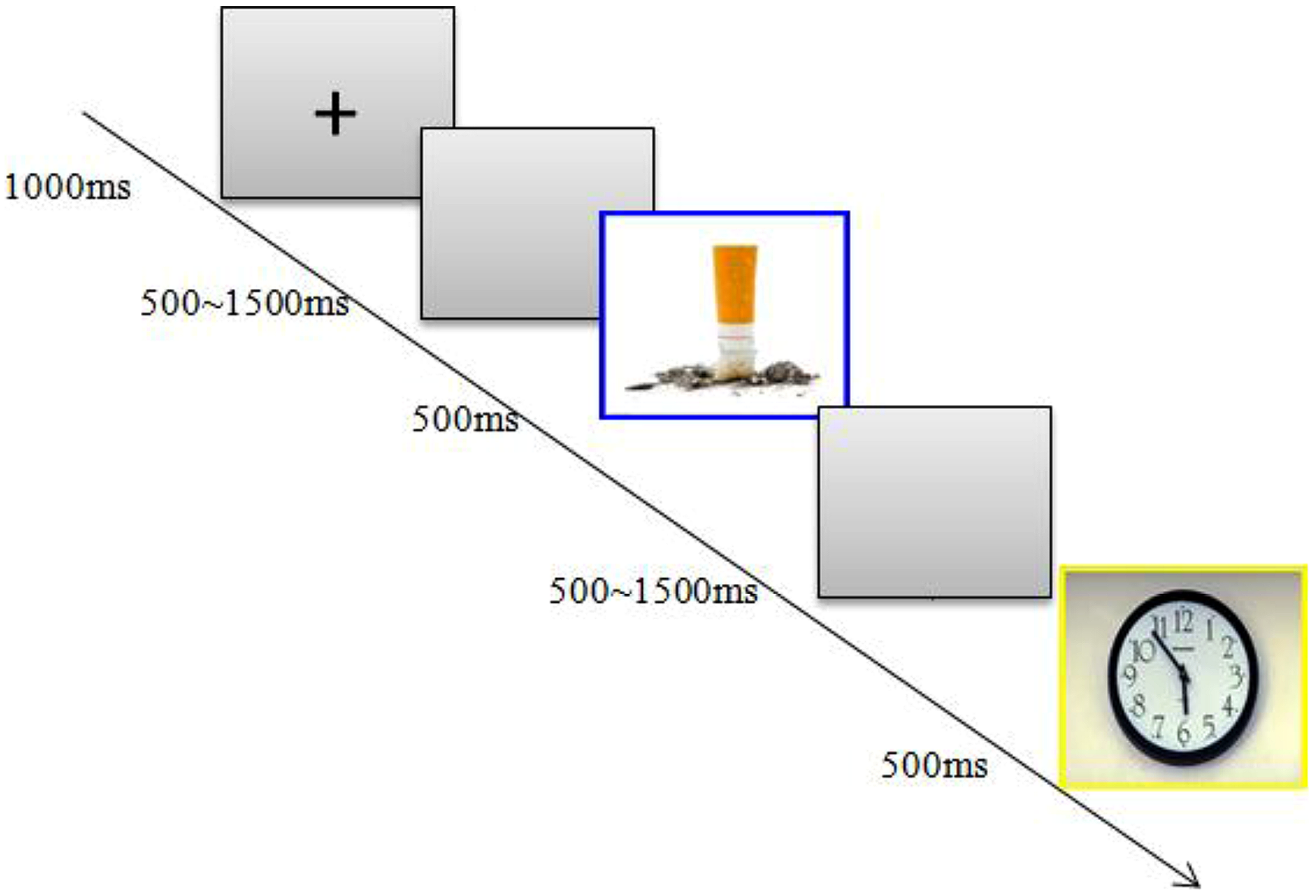
**Study design.** Participants were shown neutral pictures frequently and smoking-related pictures infrequently.

The participants were seated in an acoustically isolated room at approximately 60 cm from a computer screen. Each block began with a 1000 ms presentation of a small black cross on a gray computer screen; then, a blank screen whose duration varied randomly from 500 to 1500 ms was presented, and then, a stimulus was presented for 500 ms (see **Figure 1**). In the current study, the participants were instructed to make a standard/deviant categorization by pressing different keys as accurately and, then, as quickly as possible. Within a sequence, the order of the presentation was random, with the restriction that the deviant stimuli could only be presented twice in succession. Additionally, accurate responses to both the standard and deviant stimuli were emphasized during the task. Because the standard stimuli were presented much more frequently than were the deviant stimuli, to make a correct response to the deviant stimuli, the participants consequently had to inhibit dominate responses to the standard stimuli during the onset of the deviant stimuli.

Within each group, half the participants were instructed to press the “F” key with their index finger (as accurately and quickly as possible) if the standard picture appeared and to press the “J” key if the deviant picture appeared. For the remaining participants, the assignment of response hands was reversed for controlling the influence of response hands. The stimulus picture ceased to appear on the screen if a key was pressed or after the picture had appeared for 1000 ms. Therefore, the participants were informed that they had to respond within 1000 ms. Each response was followed by a blank screen (which appeared randomly from 500 to 1500 ms). Pre-training with 20 practice trials was conducted before the experiment to familiarize the participants with the procedure, and the pictures used during the practice were not used in the experimental block. All the participants achieved 85% accuracy during practice.

All the participants provided basic information and informed consent, completed the FTND, BDI, and BIS-11 using a paper and pencil, and then started the main experiment. The entire experiment lasted for 15 min.

## Results

The accuracy and RT for deviant and standard stimuli were analyzed to assess response inhibition. The data for less than 15% of the trials (i.e., trials for which RTs were less than 150 ms) were not considered (Meule et al., 2012). The main statistical methods in the database—repeated measures ANOVA were used. In the experimental task, the between-subjects variable was group (male light smokers vs. non-smokers), and the within-subject variable was stimulus type (deviation stimuli vs. standard stimuli).

### Accuracy

**FIGURE 2:**
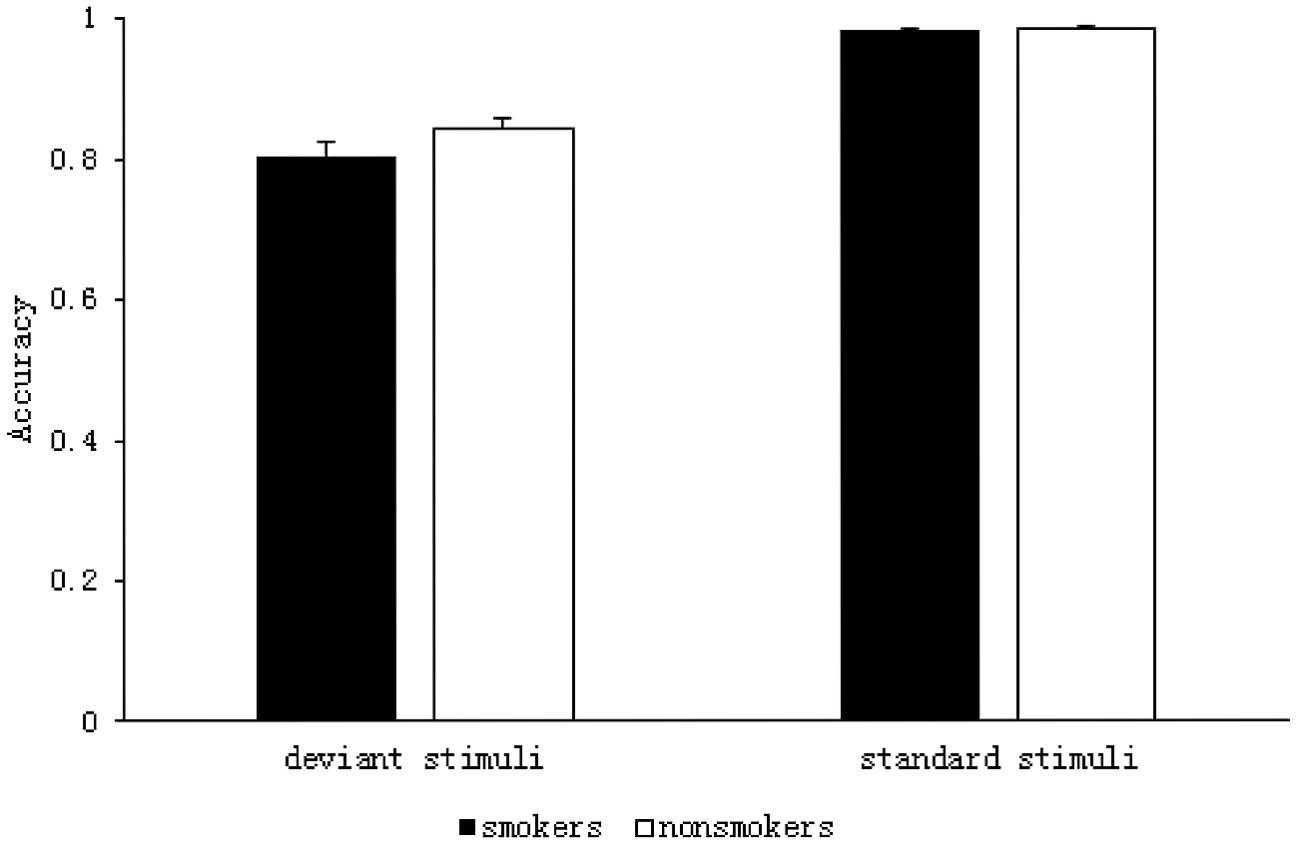
**Accuracy of smokers and non-smokers for standard and deviant stimuli**.

The results of the 2 (group: smoker, non-smoker) × 2 (stimulus type: deviant, standard) repeated measures ANOVA are shown in **Figure 2**. The main effect of group was not significant *F*(1,59) = 2.191, *p* = 0.144, η^2^= 0.036. However, there was a significant main effect of stimulus type *F*(1,59) = 181.384, *p* < 0.001, *η^2^* = 0.755; ACC was significantly lower for the deviant stimuli (*M* = 0.82, SD = 0.01) than it was for the standard stimuli (*M* = 0.99, SD = 0.001) in both the groups. There was no significant interaction between group and stimulus type *F*(1,59) = 1.941, *p* = 0.169, η^2^= 0.032.

### Reaction Time

**FIGURE 3:**
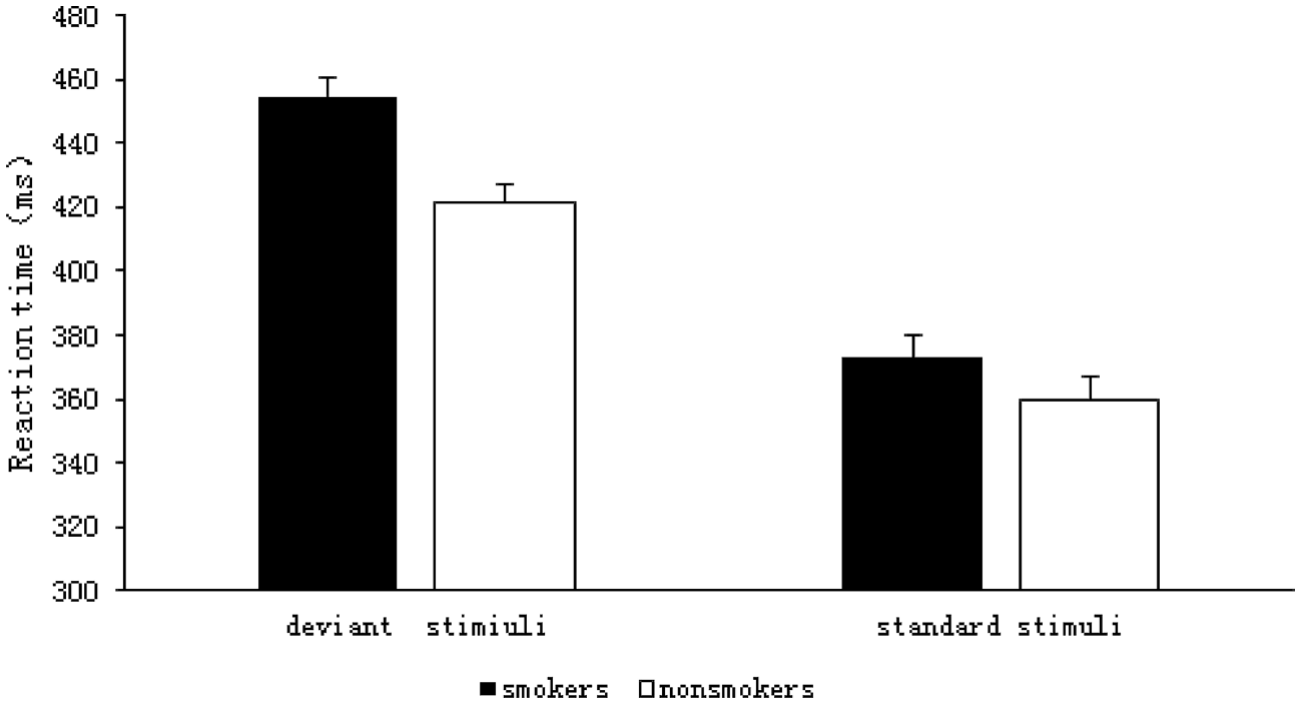
**RTs for smokers and non-smokers for standard and deviant stimuli**.

The results of the 2 (group: smoker, non-smoker) × 2 (stimulus type: deviant, standard) repeated measures ANOVA are shown in **Figure 3**. The RT analysis indicated a significant main effect of group *F*(1,59) = 8.512, *p* = 0.005, η^2^= 0.126, such that the RTs were longer for the smokers (*M* = 413.71, SD = 5.65) than they were for the non-smokers (*M* = 390.58, SD = 5.56). There was also a significant main effect of stimulus type *F*(1,59) = 228.05, *p* < 0.001, η^2^= 0.794; RTs were significantly longer for the deviant stimuli (*M* = 438.07, SD = 4.10) than they were for the standard stimuli (*M* = 366.22, SD = 5.09) in both the groups. There was a significant interaction between group and stimulus type *F*(1,59) = 4.213, *p* = 0.045, η^2^= 0.067, and a simple effect analysis showed that the RTs for deviant stimuli were significantly longer for smokers (*M* = 454.52, SD = 5.85) than they were for non-smokers (*M* = 421.62, SD = 5.75), *F*(1,59) = 16.091, *p* < 0.001, η^2^= 0.214. In contrast, the RTs for standard stimuli did not differ between the groups (smokers: *M* = 372.91, SD = 7.26; non-smokers: *M* = 359.54, SD = 7.14, *F*(1,59) = 1.723, *p* = 0.194, η^2^ = 0.028).

## Discussion

The aim of the present study was to explore the differences between male light smokers and non-smokers with regard to the ability to inhibit responses to cigarette-related cues, using the two-choice oddball paradigm. The results showed that compared to non-smokers, male light smokers have a poor ability to inhibit responses. Specifically, male smokers showed a longer delay effect for deviant cigarette-related stimuli. The results of this study are consistent with those of previous researches (Powell et al., 2004; Bekker et al., 2005; Musso et al., 2007; Billieux et al., 2010; Luijten et al., 2011). For deviant stimuli, the RTs were significantly longer for smokers than they were for non-smokers. The results indicated that cigarette-related stimuli had a greater impact on response inhibition in male light smokers than they did for response inhibition in male non-smokers. In other words, compared to non-smokers, the response inhibition of male light smokers is closely associated with cigarette-related cues.

In recent years, some studies have found that compared to controls, response inhibition ability in smokers were affected by nicotine intake (Monterosso et al., 2005; Luijten et al., 2011; Charles-Walsh et al., 2014). Smokers were further divided into light, moderate and heavy group. Compared with moderate group and heavy group, response inhibition ability in light smokers was not influenced by the nicotine. For example, some studies did not find differences in inhibitory control (measured with Go/No-Go or stop-signal tasks) between non-smokers and light smokers (5–10 cigarettes per day; Dinn et al., 2004; Reynolds et al., 2007). In addition, Robinson et al. (1992) recruited five male smokers, after 48-h abstention from tobacco product use, smoking a leading “light” category cigarette (Control 0.6 mg nicotine) and another cigarette yielding similar amount of “tar” and carbon monoxide (CO), but only 0.06 mg nicotine (Test). The electroencephalogram (EEG) were monitored before, during and after the smoking of each cigarette. The results indicated that smoking the Test cigarette had no effect on the EEG. Smoking the Control cigarette produced a significant increase in beta2 magnitude and a significant decrease in delta magnitude. This study selected male light smokers as participants, and they showed poor response inhibition for cigarette-related cues. Probably because cigarette-related cues lead to a craving for smoking, and this craving leads smokers to automatically attend to cigarette-related cues. Consequently, cognitive resources were occupied, which affected the smokers’ performance on the cognitive task (Johnsen et al., 1997; Waters and Feyerabend, 2000; Ehrman et al., 2002; Ryan, 2002; Franken, 2003). Previous studies have found that the presentation of cigarette-related cues interfered with working memory encoding (Heishman et al., 2006), thus affecting the response speed for visual stimuli (Sayette and Hufford, 1994), reading (Zwaan et al., 2000), and arithmetic tasks (Madden and Zwaan, 2001). In general, as addiction develops, the addictive substance-related stimulus becomes particularly attractive, and can grab individuals’ attention (Field and Cox, 2008). A recent study found that individuals’ attentional bias to addictive substance-related cues is positively related to decreased inhibition control in decision-making, especially when the decisions are related to the addictive substance (Field et al., 2007).

The majority of previous studies have used Go/NoGo and stop-signal tasks to show the effects of nicotine on executive function in smokers (Bekker et al., 2005; Billieux et al., 2010; Luijten et al., 2011). In recent years, researchers have also adopted the two-choice oddball paradigm to investigate response inhibition (Wang et al., 2011; Yuan et al., 2011). Here, the two-choice oddball paradigm was used to explore response inhibition for cigarette-related cues in male light smokers. Cigarette-related pictures and cigarette-unrelated pictures were used as deviant and standard stimuli, respectively. The response inhibition of smokers was compared to that of non-smokers, and so was the difference in results among the Go/No-Go, stop-signal, and two-choice oddball paradigms. Compared to Go/NoGo tasks and stop-signal tasks, in the two-choice oddball paradigm, in addition to perceptual processing, stimulus identification, response selection, and execution, deviant stimuli require the behavioral inhibition of the dominant response. In contrast, behavioral inhibition is not required for standard stimuli (Yuan et al., 2008). Therefore, regardless of whether the cues are cigarette-related or cigarette-unrelated; the RTs were significantly longer for the deviant stimuli than they were for the standard stimuli. Because of the need for response inhibition, all the participants showed a significant delay effect for deviant stimuli, but this was especially pronounced in male light smokers.

We only used the two-choice oddball paradigm to explore male light smokers’ behavioral ability to inhibit responses to cigarette-related cues. The neurophysiological mechanism underlying impaired response inhibition for cigarette-related cues in smokers remains unclear. Luijten et al. (2011) compared ERP components of 19 moderate smokers and 20 non-smokers, in a Go/NoGo task. The results revealed that moderate smokers’ NoGo-N2 amplitude was significantly lower than that of non-smokers, and the two groups showed no significant difference in NoGo-P3. Studies have found that Go/NoGo tasks activate some areas of the left side of the brain, such as the anterior cingulate cortex and pre-supplementary motor areas that are associated with tasks involving intense exercise and behavior choice. This suggests that the inhibition observed in the case of Go/NoGo tasks was mainly due to behavior choice and preparation. Therefore, rather than using Go/NoGo tasks, in the future, researchers should use ERPs in the context of the two-choice oddball paradigm, which will enable them to measure electrical brain activity related to behavioral control, and should examine the differences between ERPs for standard stimuli and those for deviant stimuli (Wei et al., 2002; Yuan et al., 2008). In addition, it may be possible to observe differences in the underlying brain mechanism of smokers and non-smokers in the two-choice oddball paradigm, using functional magnetic resonance imaging. Because only male light smokers were tested in this study, future studies should explore response inhibition in female smokers, and examine gender-based differences in the ability to inhibit responses to cigarette-related cues. Here, we can make a comparison between light smokers and non-smokers; Another important question for future research is whether differences in response inhibition vary on the basis of degree of smoking (moderate, and heavy) when studied in the context of the two-choice oddball paradigm.

## Conflict of Interest Statement

The authors declare that the research was conducted in the absence of any commercial or financial relationships that could be construed as a potential conflict of interest.
